# A Novel Passive Wireless Sensing Method for Concrete Chloride Ion Concentration Monitoring

**DOI:** 10.3390/s17122871

**Published:** 2017-12-11

**Authors:** Shuangxi Zhou, Wei Sheng, Fangming Deng, Xiang Wu, Zhihui Fu

**Affiliations:** 1 School of Civil Engineering and Architecture, East China Jiaotong University, Nanchang 330013, China; zhoushuangxi@ecjtu.jx.cn (S.Z.); 2016018081402008@ecjtu.jx.cn (W.S.); 2 School of Electrical and Automation Engineering, East China Jiaotong University, Nanchang 330013, China; 2464@ecjtu.jx.cn (X.W.); fuzhihui@ecjtu.jx.cn (Z.F.)

**Keywords:** RFID technology, wireless measuring, concrete chloride ion concentration, monitoring

## Abstract

In this paper, a novel approach for concrete chloride ion concentration measuring based on passive and wireless sensor tag is proposed. The chloride ion sensor based on RFID communication protocol is consisting of an energy harvesting and management circuit, a low dropout voltage regulator, a MCU, a RFID tag chip and a pair of electrodes. The proposed sensor harvests energy radiated by the RFID reader to power its circuitry. To improve the stability of power supply, a three-stage boost rectifier is customized to rectify the harvested power into dc power and step-up the voltage. Since the measured data is wirelessly transmitted, it contains miscellaneous noises which would decrease the accuracy of measuring. Thus, in this paper, the wavelet denoising method is adopted to denoise the raw data. Besides, a monitoring software is developed to display the measurement results in real-time. The measurement results indicate that the proposed passive sensor tag can achieve a reliable communication distance of 16.3 m and can reliably measure the chloride ion concentration in concrete.

## 1. Introduction

The chloride in concrete is one of the major reasons of deterioration of concrete structures [1,2,3,4,5]. To avoid the corrosion-induced damage, effective prevention, including monitoring of the aggressiveness of the environment is of great significance. Therefore, the monitoring for chloride ion concentration plays a vital role in estimating its service life and maintenance cycle.

The commonly used procedure includes collecting the concrete powder samples at various depths of which are then used to quantify the total chloride content [6]. Although this approach has been well-established, the disadvantages of its being invasive and time-cost limit it being widely employed. The probes for continuous monitoring are another approach to measure the chloride ion concentration [7]. They need to be embedded in the concrete before casting, but it can only realize practically instantaneous measurement of chloride content. The method using the Ag/AgCl electrode to measure the chloride ion content also garners great interest [8,9,10,11]. However, this method is an unreliable intermittent measurement. Montemor et al. [7] presented a non-destructive chloride-sensitive sensor, but it requires high implementation costs. Hence, a non-invasive and cost-effective method is still urgently demanded.

In recent years, various kinds of wireless sensors have been widely employed in industrial fields due to their fast deployment and wireless features. Generally, the wireless sensors utilize technologies of ZigBee, Bluetooth and wireless local area networks to realize wireless communication [12,13,14]. To date, these wireless sensors are generally adopted to establish wireless sensor networks for environment monitoring [15]. However, the high power consumption, large device size, and low flexibility limit its popularity. Nowadays, wireless sensors based on passive Radio Frequency Identification (RFID) technology have arisen great interest due to their low power consumption and high flexibility [16]. Most RFID-based sensors operate on ultra-high frequency (UHF) to realize long communication distance and fast transmission speed. The RFID-based sensor is capable of communicating with the reader based on zero-powered backscatter mechanism, resulting in the advantages of simple architecture, low power and low cost compared with these technologies discussed above. Since the chloride ion concentration is one of the most important parameters in evaluating the concrete’s performance, the passive wireless sensor based on RFID communication protocol is promising to be widely employed.

The passive wireless sensors based on UHF RFID technology convert the received RF signals from RFID reader into stable DC supply voltage for their own circuits. A customized power harvesting and management circuit is proposed to utilize the received power with high efficiency. An Ag/AgCl chloride ion selective electrode is employed to perform as a sensor to detect the potential of the concrete, which can reflect the chloride ion concentration of concrete.

This paper introduces a novel passive wireless sensor for long-time and long-range chloride ion concentration sensing application. This wireless sensor employs the backscattering scheme of RFID technology for passive operating and Ag/AgCl chloride ion selective electrode for chloride ion concentration measuring. A customized energy harvesting and management circuit is proposed to improve the efficiency of RF power utilization.

## 2. Materials and Methods

### 2.1. RFID Sensor Technology

**Figure 1:**
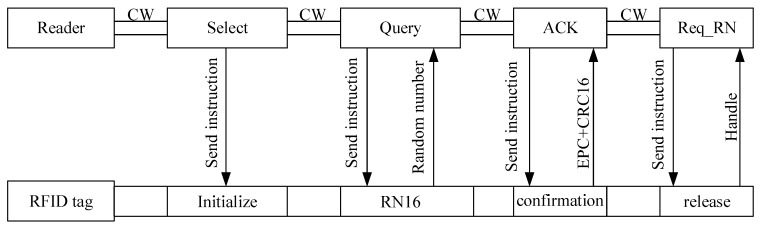
Schematic diagram of radio frequency communication.

The RFID system generally consists of RFID tag and RFID reader. Figure 1 shows the basic protocol. The RFID tag returns data to the reader based on backscatter mechanism, and the communication between the tag and reader follows the EPC Class-1 Generation-2 protocol. Within the communication distance, the reader is sent select instruction by carrier wave to the tag to change the tag’s status. Then the Query instruction is sent by the reader to the tag and the tag response with RN16. After that, the reader sends ACK instruction to obtain the ID message and CRC code. Finally, Req_RN is sent to acquire the Handle response.

**Figure 2:**
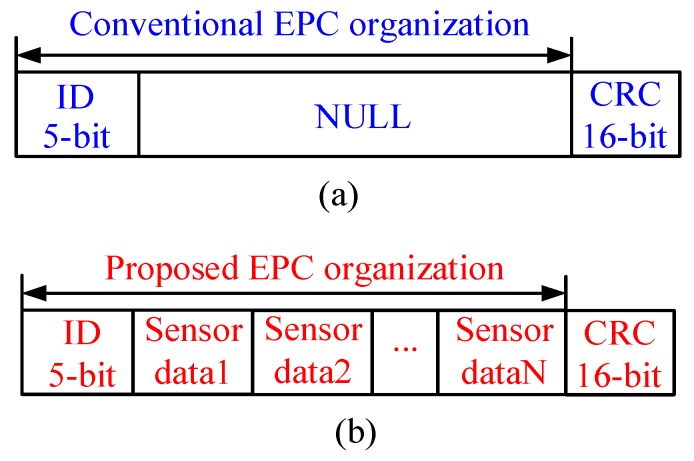
EPC area coding scheme of RF Tag chip: (**a**) conventional organization strategy; (**b**) proposed organization strategy.

Figure 2 illustrates the memory organization comparison between conventional RFID tag and the proposed tag in this paper. A prominent merit of the proposed tag is the capability of its memory being accessed by the MCU via I^2^C interface. Generally, the (Electronic Product Code) EPC area in the tag memory can be organized by users, thus in this paper, the measured sensor data are stored in the EPC area. ID message is employed to identify the tag, and cyclic redundancy check (CRC) is adopted to check the integrity and accuracy of data transmission.

### 2.2. RFID Sensor Tag Topology

**Figure 3:**
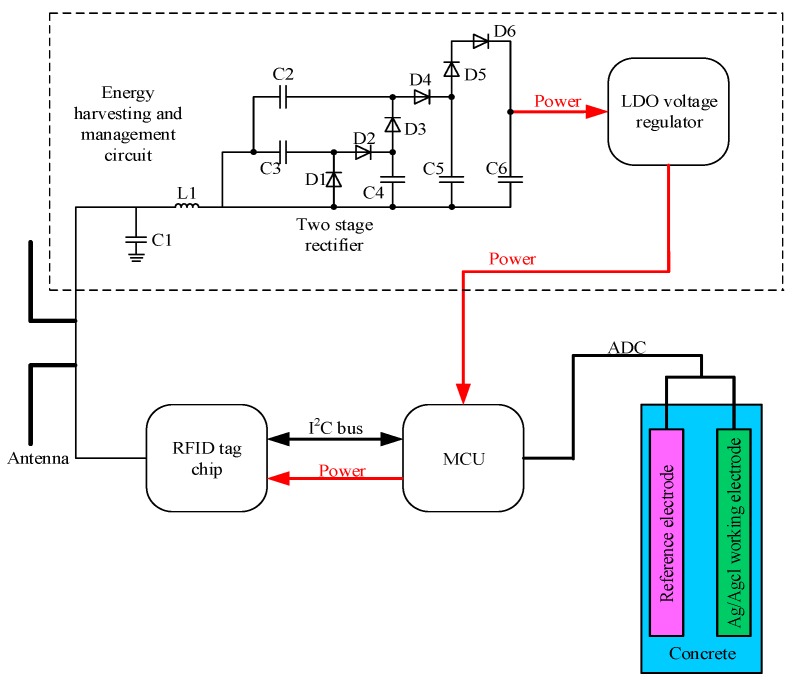
The block diagram of the proposed RFID sensor tag.

Figure 3 draws the block diagram of the exploited RFID sensor tag (hereafter called tag). The proposed tag operates in full passive mode with ultra-high frequency (915 MHz), a 50-Ω microstrip antenna is employed for both communication and energy harvesting. A LC impedance-matching network consisting of a RF inductor (L1) and a ceramic trimmer capacitor (C1) is adopted to realize maximum power transfer efficiency. Then, a three-stage boost rectifier is used to convert the received UHF RFID energy by the tag to dc power and step-up the output voltage. When the input voltage of the boost rectifier is 0.35 V or higher, it can generate a 2.4 V output voltage, which would be the input of a 1.8 V low dropout (LDO) voltage regulator. The SMS7630 zero-bias Schottky diodes (D1–D6) are selected as rectifying devices due to their high sensitivity at the operating frequency of the tag.

The RFID chip adopted in this paper is Monza X-8K which can provide a UHF air interface as well as an I^2^C interface. This design strategy adopts an ultra-low power MCU MSP430 MCU which can provide 64 kB RAM and a 12-bit 200-ksps Analog-to-Digital Converter (ADC). The MCU consumes 100 μA in measurement mode. The sensor employed to monitor the chloride ions consists of reference electrode and Ag/Agcl working electrode; both of the electrodes are immersed into the concrete. These two electrodes will generate a voltage difference, and its value would change in different chloride concentrations. The Ag used in this paper has a diameter of 1mm and 99.99% purity, which is pasted with a thick layer of Ag/Agcl paste. A thick layer of paste is essential to the long lifetime of the tag, thus in this paper a 600 μm thick layer of paste is employed.

### 2.3. Tag Antenna Design

**Figure 4:**
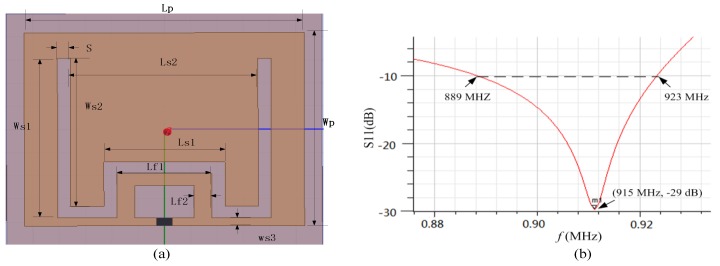
Proposed patch antenna: (**a**) antenna design; (**b**) return-loss in air.

**Figure 5:**
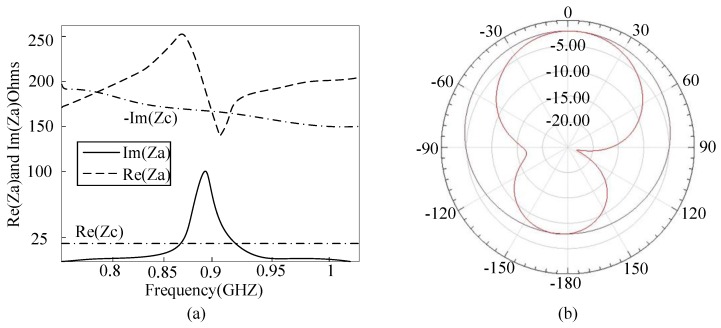
(**a**) Simulated impedance match between tag and antenna; (**b**) Simulated pattern radiation of the tag antenna.

Microstrip antenna has prior performance in penetrating concrete compared to the dipole antenna [17,18]. In conventional microstrip antenna design strategy, the rectangular length is equal to half of its electrical length, resulting in a large tag size. Thus, a miniaturized microstrip antenna utilizing the embedded short stub and U-type slot (shown in Figure 4a) is proposed to overcome the abovementioned drawback [19]. The impedance of tag (Zc) is 19-j172 Ω at 915 MHz. The antenna is made of copper traces with the thickness of 0.035 mm. The antenna impedance can be manually set by adjusting the values of Lf1 and Lf2, since the current in the short-circuited stub is the strongest. The stub inside the radiation patch can lead to a decrease in antenna size. In addition, since the current can flow in the groove, it leads to an increase in the length of the current path, so as to achieve the purpose of volume miniaturization. The return loss of the antenna is illustrated in Figure 4b. The return loss in the frequency of 915 MHz is −29 dB and the bandwidth is less than 10 dB from 889 to 923 MHz. The detailed parameters of the proposed patch antenna are listed in Table 1. The antenna impedance and radiation pattern can be obtained by simulation in HFSS. The substrate of antenna is FR4; dielectric constant and thickness is 4.5 and 1.5 mm, respectively. The antenna impedance Za matches well to the chip at the resonance frequency of 915 MHz (shown in Figure 5a). The proposed sensor tag is buried in 20 mm of thick concrete, and the measured antenna gain is −3.2 dB with efficiency of 37.2% (shown in Figure 5b).

### 2.4. Design of Chloride Ion Sensor

Ag/AgCl belongs to second-class electrode, which consists of metal and metal insoluble salt. Since argentic chloride has small solubility in solution, it can maintain its electrode potential in a long term. Moreover, it can decrease the possibility of solution being fouled. The corresponding electrode reaction is [7]:

(1) $$\mathrm{AgCl}+e^{-}\to \mathrm{Ag}+{\mathrm{Cl}}^{-}$$

The potential of Ag/AgCl electrode (*E*) can be calculated by:

(2) $$E=E^{s}-\frac{RT}{F}\ln a_{Cl^{-}}$$

where *E^s^* is the standard potential of Ag/AgCl, *R* is ideal gas constant, *T* is the environmental temperature, *F* is the Faraday constant, and $a_{Cl^{-}}$ is the activity quotient of ${\mathrm{Cl}}^{-}$ in the environment. Generally, the solution has low concentration, which means the activity quotient keeps almost constant, so the activity quotient can be replaced by concentration. In this way, Equation (2) can be simplified as:

(3) $$E=E^{s}-59.21\mathrm{lg}[{\mathrm{Cl}}^{-}]$$

**Figure 6:**
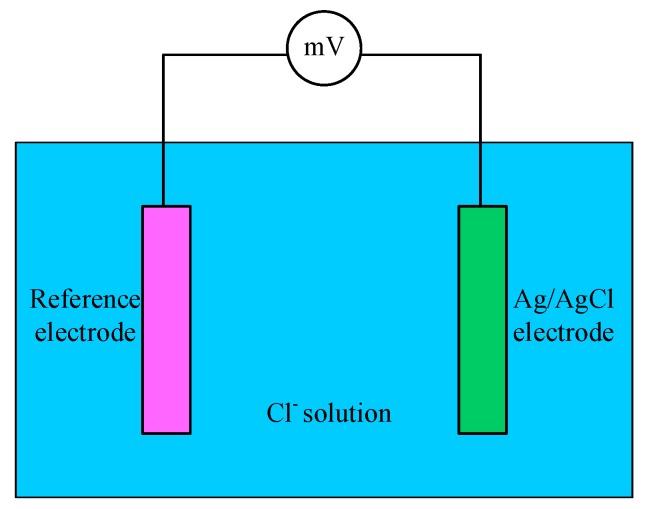
Principle of chloride concentration detection.

From Equation (3) it can be seen that the electrode potential is linearly related to the negative logarithm of chloride concentration in the solution. Therefore, immersing the electrode into the concrete and then the corresponding potential can be obtained. According the potential value and calibration curve, the chloride concentration can be acquired. Figure 6 shows the principle of chloride concentration detection using Ag/AgCl electrode.

The Ag/AgCl electrode can be obtained by electrochemical deposition technique. The specific steps are as follows: firstly, the Ag wire is polished by water proof abrasive papers. Then, the polished Ag is cleaned using acetone, anhydrous ethanol, and distilled water respectively. Finally, the Ag is used as anode and Pt wire is employed as cathode and then the Ag wire is be coated by a layer of AgCl paste.

**Figure 7:**
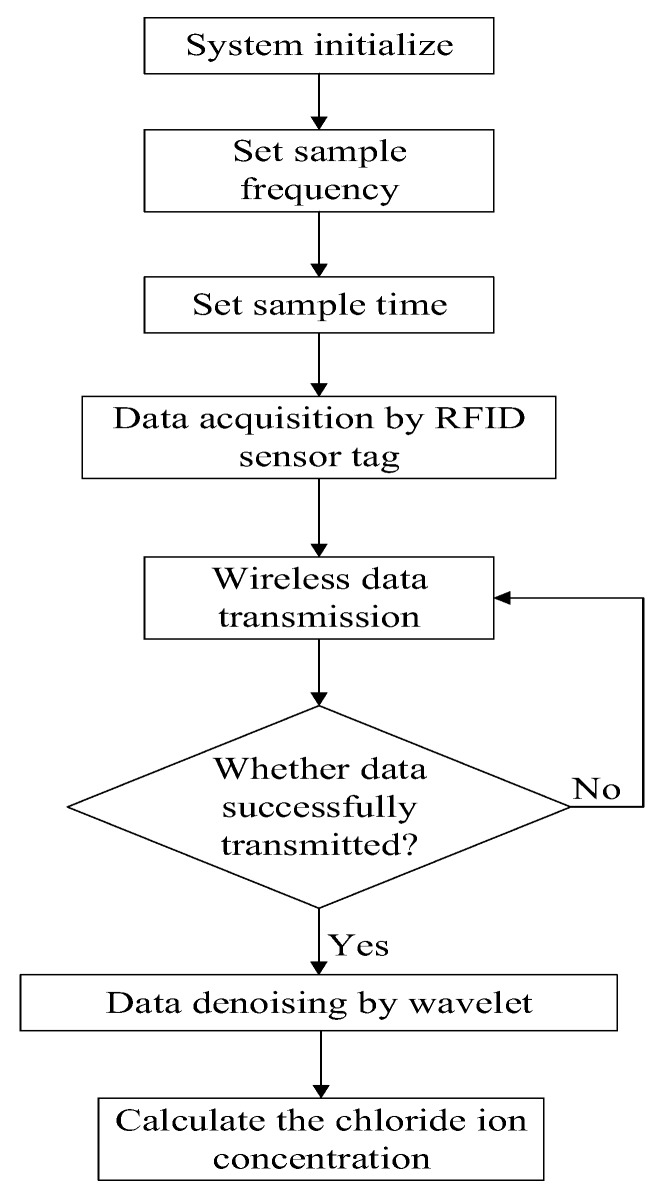
The whole measuring flow.

The whole measuring flow is shown in Figure 7. The sample frequency and sample time is pre-set, and the data is acquired by the proposed RFID sensor tag and then wirelessly transmitted.

### 2.5. Wavelet Denoising

Since the measured data is wirelessly transmitted, it contains miscellaneous noises which would decrease the accuracy of monitoring. Thus, it is important to extract the original data from the mixed data. Recently, several denoising methods have been introduced [20,21,22], among which the wavelet denoising method is most widely employed due to its satisfactory performance and high operation speed. Thus, in this paper, wavelet denoising method is employed to address this concern.

Assume the transmitted data consists of two parts:

(4) r(c)=x(c)+n(c)

where *r*(*c*) is the mixed data, *x*(*c*) is the original data and *n*(*c*) is the noise. Assume that Z is an integer set, {*V_t_*}, t∈Z is an orthogonal multiresolution analysis, and {*W_t_*}, t∈Z is the corresponding wavelet space. The signal *r*(*c*) is projected to the wavelet space on *V_t_* is:

(5) $$P_{V_{t}}=P_{V_{t+1}}+P_{w_{t+1}}=\sum_{i\in Z}c_{t+1}^{i}\varphi_{t+1,i}+\sum_{i\in Z}d_{t+1}^{i}\psi_{t+1,i}$$

where $P_{V_{t+1}}$ and $P_{w_{t+1}}$ represents the projections of *r*(*c*) on *V_t_* and *W_t_* with resolution of 2^*t*+1^, respectively; $c_{t+1}^{i}$ and $d_{t+1}^{i}$ are the scaling coefficient and wavelet coefficient of *r*(*c*) with resolution of 2^*t*+1^, respectively. Hence, the *c_t+_*_1_ and *d_t+_*_1_ denote the approximations and details of *r*(*c*) with resolution of 2^*t*+1^, respectively. The {*V_t_*}, t∈Z can be correspondingly decomposed into:

(6) $$\begin{array}{ll} V_{t}=W_{t+1}⊕V_{t+1} & =W_{t+1}⊕(W_{t+2}⊕V_{t+2})\\ & =W_{t+1}⊕W_{t+2}⊕(W_{t+3}⊕V_{t+3})\\ & =W_{t+1}⊕W_{t+2}⊕W_{t+3}⊕\cdots \end{array}$$

The discrete approximation coefficients and detail coefficients are generated by employing multilevel wavelet decomposition. The detail coefficients which have small absolute value are treated as noise. In the conventional wavelet denoising approach, the detail coefficients below the threshold (pre-set) are set to zero and reconstructed by the original data by employing the rest coefficients. Universal threshold, rigorous threshold, heursure threshold, and minimax threshold are four commonly used thresholds in the wavelet denoising method. Generally, single threshold method may result in local optimal phenomenon. Thus, in this paper, the universal threshold and minimax threshold are adopted together.

The universal threshold (also known as VisuShrink) employs the length of data to generate a threshold, it can be calculated by the following equation:

(7) $${\mathrm{Threshold}}_{\mathrm{universal}}=\sigma \sqrt{2\log n}$$

where *σ* represents the compensation factor and *n* is the length of data.

The minimax threshold method is an optimal threshold, which is obtained by minimizing the constant term on the upper bound of the risk in terms of the function estimation. The threshold is computed as follows:

(8) $${\mathrm{Threshold}}_{\mathrm{minimax}}=\alpha +\beta \times \frac{\log(n)}{\log(2)}$$

where *α* and *β* are the coefficients that are commonly set to 0.4013 and 0.1798, respectively.

From the equations above, it can be deduced that the value of minimax threshold is lower than that of universal threshold. The universal threshold method can effectively weaken the strong noise, and the minimax threshold method can effectively remove the weak noise. Thus, in this paper the universal threshold method is firstly performed and then the minimax threshold method is performed.

## 3. Results and Discussion

**Figure 8:**
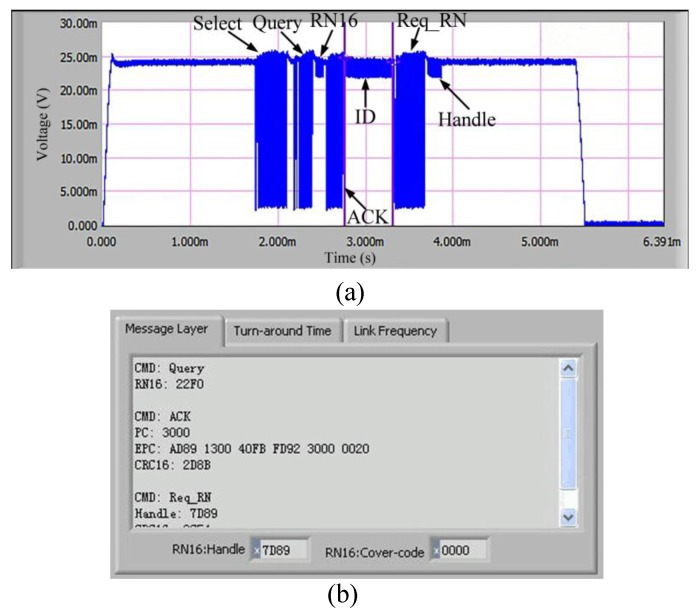
Overall communication flow: (**a**) corresponding waveform; (**b**) corresponding data.

The communication performance of the introduced tag should be firstly evaluated. The operating frequency of the tag is 915 MHz; the distance between the tag and the reader are 1.5 m, and the radiation power of the reader is 4 W. The measured communication flow is shown in Figure 8. Firstly, the tester sends Select command to the tag. After 5—6 Tari, the reader sends Query command to the tag and obtain a response of RN16. Next, the reader sends ACK command to obtain the tag’s ID message as well as the measured data. Finally, the reader sends Req_RN command to acquire the Handle response. The digital data in the command are shown in Table 2.

**Figure 9:**
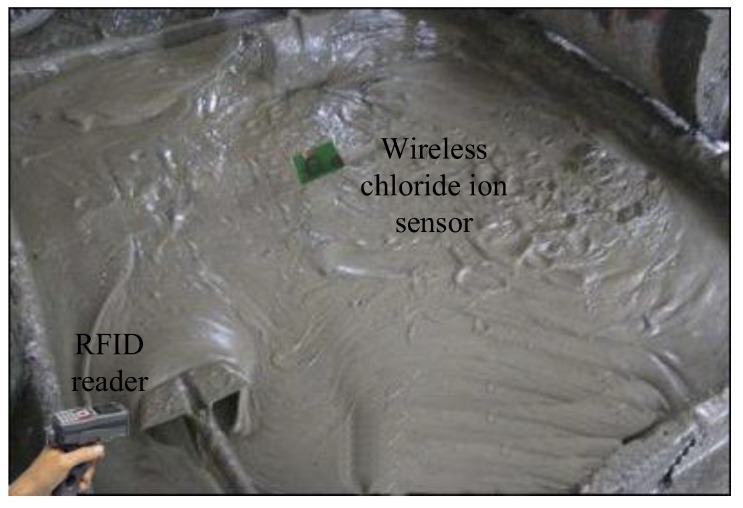
Test environment.

**Figure 10:**
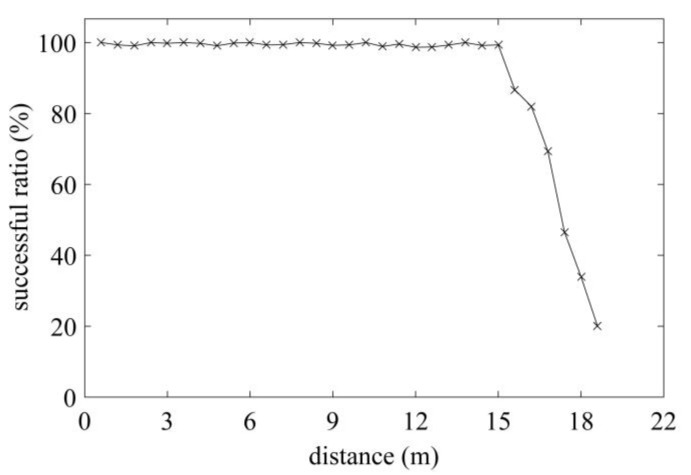
Measured successful ratio under different distance.

The maximum communication distance determines the performance of the exploited tag, thus, a standard RFID Gen2 tester operating at 915 MHz is employed to measure the maximum communication distance on-site (shown in Figure 9). The R1 reader [23] and the tag are placed on the line of sight. The distance between the reader and the tag is increased in steps of 0.2 m, and 1500 attempts are instructed to communicate with the tag at each distance. The successful communication ratio at different distances is recorded in Figure 10. Based on the analysis in [24], the successful communication ratio should larger than 80% to guarantee reliable communication performance. In Figure 10, the ratio remains above 80% until 16.3 m, which means the maximum distance in read is 16.3 m; such a result suits our expectation well. The measured minimum communication distance is 10cm in the test scenario.

**Figure 11:**
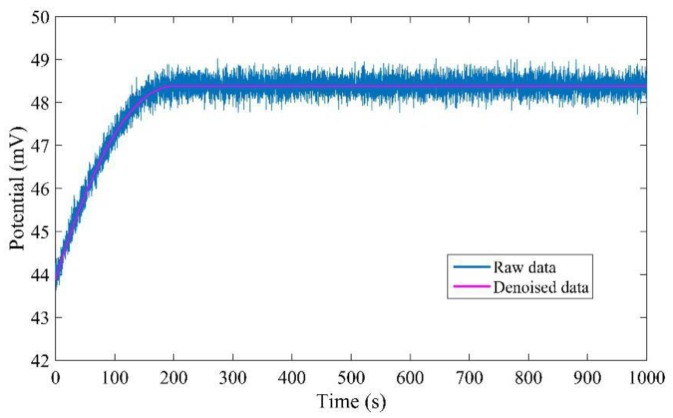
Comparison of raw data and denoised data.

The measured data are denoised twice by the wavelet denoising method with different thresholds. Firstly, they are processed by the wavelet denoising method with universal threshold, and then they are processed by the wavelet denoising method with minimax threshold. The raw data and denoised data are shown in Figure 11. It is obvious that the proposed denoised method can effectively remove all the noise.

**Figure 12:**
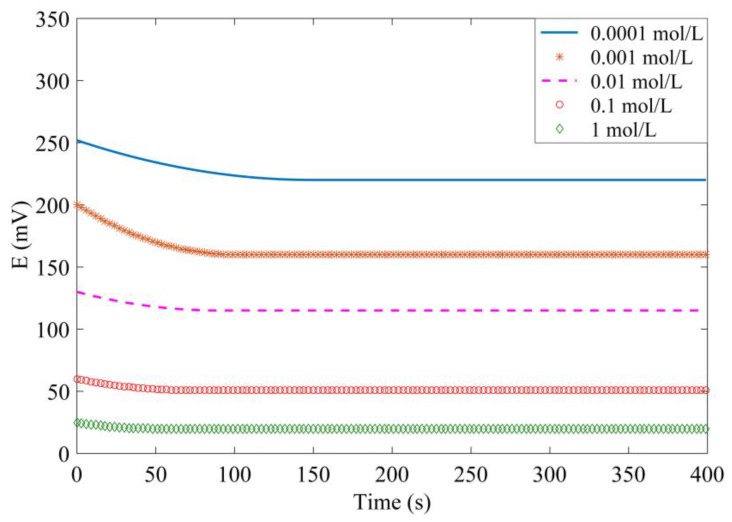
Measured potential variation over time.

The experiment is carried out in concrete mixed with NaCl solution in concentrations of 0.0001, 0.001, 0.01, 0.1, and 1.0 mol/L. Figure 12 shows the potential variation over 400 s. It can be seen that the response time is different in all of these five solutions, but they all achieve the stable value within 150 s. Moreover, the response time is longer in a dilute solution. The experimental results indicate that the Ag/AgCl electrode has high sensitivity to the chloride concentration and has a high response speed.

**Figure 13:**
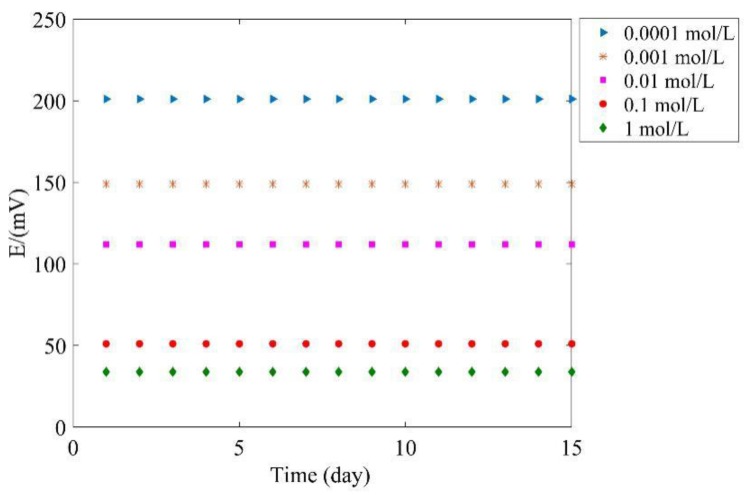
Long term stability.

In order to evaluate the long-term potential stability of the proposed tag, the potential is measured every day and lasts 15 days; the results are shown in Figure 13. As seen, the potential keeps stable during the test with maximum fluctuation within 5 mV. The results prove that the proposed tag has persuasive performance in long term stability.

**Figure 14:**
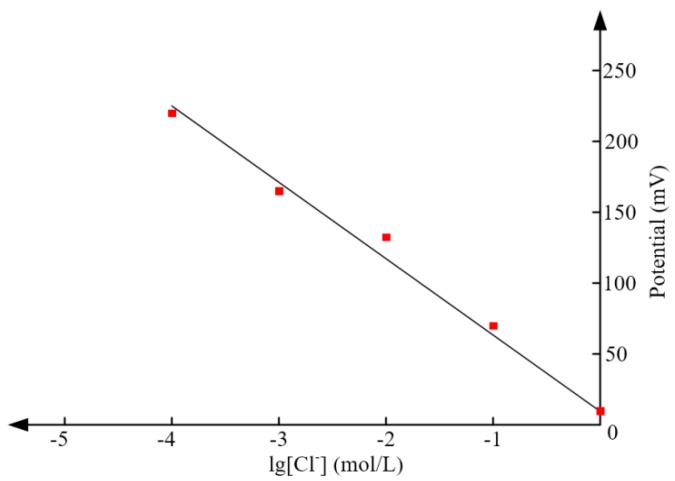
The relationship between electrode potential and chloride concentration.

The theoretical relation between the chloride concentration and potential has been obtained in Equation (3). To validate this equation, the potential of Ag/AgCl electrode with respect to Pt in solutions of different concentration is tested. Figure 14 shows the measured results and the potential is a steady value reached 3 min after the electrode was immersed in the solution. From Figure 14, it can be seen that the potential in solution of 0.001 mol/L is 153.48 mV, the corresponding linear fitting equation is *y* = −47.83*x* + 10. The calculated slope is −47.83, which is close to the theoretical value (−59.211). It can be deduced that the proposed tag has high sensitivity in measuring chloride concentration.

The selection of reference electrode is also significant to the performance of tag. The conventional reference electrode is calomel electrode, but it is inconvenient to be immersed in the concrete since it contains solution. Thus, in this paper the reference electrode is made of Pt wire, which has a simple structure and can be easily immersed in the concrete. To evaluate the performance of the adopted reference electrode, the Pt reference electrode couple, calomel reference electrode couple and Ag/AgC1 electrode couple are inserted into the Sodium Chloride Solution and saturated calcium hydroxide solution which is mixed with saturated sodium chloride solution, respectively. Then the relative potential shift of different reference electrodes can be obtained. The measured results are listed in Table 3. It is clear that the maximum relative potential shifts of Pt reference electrode in these two solutions are all smaller than 3 mV, which indicates acceptable stability.

To further test the performance of the proposed tag, the commercial portable chlorine ion concentration tester (TR-C1 2501) is employed to measure the chlorine ion concentration in concrete as reference. During the test, the temperature is 20 °C, and the concentration is measured every two hours; the results are listed in Table 4. It can be seen that the results measured by the tag have high similarity with the results measured by the commercial tester. Thus, it can be deduced that the proposed tag has persuasive performance.

To better show the priorities of the proposed sensor tag, its performance has been compared with other wireless sensors that are employed for chloride ion measurement; the results are shown in Table 5. It can be seen that the proposed sensor tag in this paper has advantages of low cost, more information in memory, good anti-interferences performance, large communication range and high accuracy.

## 4. Conclusions

A novel passive and wireless method to measure the chloride ion concentration in concrete is proposed in this paper. The proposed chloride ion sensor includes an energy harvesting and management circuit, a low dropout voltage regulator, a MCU, a RFID tag chip and a pair of electrodes operating in ultra-high frequency based on RFID technology. The proposed sensor utilizes RF power from the reader as power supply. A three-stage boost rectifier is customized to rectify the harvested power into dc power and step-up the voltage. The chloride ion concentration can be obtained from the potential difference between these two electrodes which are immersed in the concrete. Owing to the satisfactory performance in penetrating concrete, microstrip antenna is employed for both communication and energy harvesting. The wavelet denoising method is adopted to denoise the raw data since the measured data contains miscellaneous noises. Moreover, a monitoring software is developed to display the measurement results in real-time. The experimental results show that the proposed passive sensor tag can achieve a reliable communication distance of 16.3 m and can reliably measure the chloride ion concentration in concrete.

## Figures and Tables

**Table 1:** Design parameters of the patch antenna.

Wp	Lp	Ls1	Ls2	Ws1	Ws2	Ws3	Lf1	Lf2	S
42 mm	53 mm	23 mm	35 mm	32 mm	29.5 mm	1.7 mm	15 mm	3 mm	2.5 mm

**Table 2:** The digital data inside the commands.

Command	RN16	PC	EPC	CRC16	Handle
Digital data	22F0	3000	AD89 1300 40FB FD92 3000 0020	2D8B	7D89

**Table 3:** Maximum relative potential shift of different reference electrodes.

Electrodes	Time (min)	Sodium Chloride Solution (mV)	Sodium Chloride Solution + Saturated Calcium Hydroxide Solution (mV)
Pt	0–2, 2–4, 4–6	2.7, 1.0, 0.6	2.1, 1.2, 0.2
Ag/AgCl	0–2, 2–4, 4–6	0.5, 0.6, 0.2	0.4, 0.5, 0.3
Calomel electrode	0–2, 2–4, 4–6	0.4, 0.2, 0.3	0.3, 0.4, 0.3

**Table 4:** Results of measured potential.

Time (h)	Potential of Proposed Tag (mV)	Potential of TR-C1 2501 (mV)
0	47.8	49.9
2	47.9	49.9
4	48.2	50
6	50.1	50
8	49.3	50
10	47.9	50
12	48.6	50.1
14	48.1	49.8
16	48.7	50
18	49.0	50.1
20	48.5	49.9
22	47.9	50
24	50.1	49.8

**Table 5:** Performances comparison of wireless chloride ion sensors.

Reported Sensor	Cost	Memory	Anti-Interferences Performance	Range (m)	Accuracy
[25]	high	Chloride ion concentration	poor	/	high
[26]	low	Chloride ion concentration	good	/	medium
[27]	high	Multi-ions concentration	poor	/	high
This work	low	Chloride ion concentration and ID message	good	16.3	high
